# Examination of the toxicity of a new group of *Karenia papilionacea* isolated from the Yellow Sea, China, to multiple species of marine aquatic animals

**DOI:** 10.3389/fmicb.2025.1687096

**Published:** 2025-11-06

**Authors:** Qiantong Chen, Menghan Gao, Ning Zhang, Lang Li, Yingyi Fan, Jiali Zhao, Xintong Xu, Zhe Tao, Yunyan Deng, Yulei Zhang, Feng Li, Siheng Lin, Ying Zhong Tang, Zhangxi Hu

**Affiliations:** 1 Guangdong Provincial Key Laboratory of Aquatic Animal Disease Control and Healthy Culture, Laboratory of Marine Ecology and Aquaculture Environment of Zhanjiang, College of Fisheries, Guangdong Ocean University, Zhanjiang, China; 2 Guangxi Key Laboratory of Marine Environmental Science, Guangxi Academy of Marine Sciences, Guangxi Academy of Sciences, Nanning, China; 3 CAS Key Laboratory of Marine Ecology and Environmental Sciences, Institute of Oceanology, Chinese Academy of Sciences, Qingdao, China; 4 Department of Biological Sciences and Biotechnology, Minnan Normal University, Zhangzhou, China

**Keywords:** *Karenia papilionacea*, morphology, molecular phylogeny, toxicity, marine animals

## Abstract

**Introduction:**

The dinoflagellate genus *Karenia* G. Hansen and Moestrup is notorious for forming harmful algal blooms (HABs), most of which can produce a variety of potent toxins (e.g., brevetoxins), killing fish and other aquatic animals above a certain cell density. Among the 11 currently accepted *Karenia* species, more than half of which are toxic, 8 species (*K. bicuneiformis*, *K. brevis*, *K. brevisulcata*, *K. hui, K. longicanalis, K. mikimotoi, K. papilionacea*, and *K. selliformis*) have been reported or described in Chinese coastal waters. Among these, *K. papilionacea* is globally distributed, with records in Asia, Europe, America, and Oceania. In China, it occurs in the East and South China Seas, though its morphological characterization and toxicology have not been well documented.

**Methods:**

In this study, we established a clonal culture of *Karenia papilionacea* through single-cell isolation from the coast of Qingdao (belonging to the Yellow Sea), China, and characterized its morphology using light microscopy (LM) and scanning electron microscopy (SEM), as well as its phylogeny based on large subunit (LSU) rDNA sequences. More importantly, we characterized the impact of *K. papilionacea* culture on brine shrimp egg hatching, as well as its toxicity to marine animals (rotifers, brine shrimp, and finfish) using laboratory bioassays.

**Results:**

We observed the typical diagnostic features of *K. papilionacea*. In phylogenetic trees inferred using Bayesian inference (BI) and maximum likelihood (ML) techniques, the Yellow Sea strain branched together with other entities of *K. papilionacea*, but formed a new group, which is different from other strains reported in the East and South China Seas. The genetic distances among our strain of *K. papilionacea* and other isolates ranged from 0.002 to 0.011, corresponding to 6–23 base differences. The Yellow Sea strain exhibited significant lethal effects on rotifer, brine shrimp, and finfish, but had a minor impact on the hatching success of brine shrimp eggs.

**Discussion:**

This study reports *K. papilionacea* in northern China for the first time, expanding the known distribution range of this toxic HAB-forming species along the Chinese coast. Our findings establish a foundation for monitoring and risk assessment of *K. papilionacea* in Chinese coastal waters and advance fundamental ecological knowledge of this toxic species. Future studies are needed to characterize toxins produced by geographical strains of *K. papilionacea*.

## 1 Introduction

Over the past half-century, the global incidence of harmful algal blooms (HABs) in estuarine and coastal ecosystems has exhibited a concerning upward trend, marked by increased frequency, greater severity, and prolonged duration (Anderson et al., 2021a; Anderson et al., 2021b; Sakamoto et al., 2021; Dai et al., 2023; Yu et al., 2023; Hu et al., 2024; Su et al., 2025). These blooms have been associated with substantial ecological impacts, including wildlife mortality, human health risks through toxin exposure, large-scale ecosystem disruptions, and considerable socioeconomic consequences across affected regions (Richlen et al., 2010; Brunson et al., 2018; Kouakou and Poder, 2019; Heil and Muni-Morgan, 2021; Yan et al., 2022). Notably, dinoflagellates constitute approximately 40% of all HAB-forming species worldwide (Jeong et al., 2021) and are responsible for approximately 75% of global HAB events (Smayda, 1997). There is an increasing trend that more and more novel toxic and harmful species have been described, which cause HABs suddenly and draw great attention from the research community (Benico et al., 2020; Cen et al., 2020; Gu et al., 2022; Hu et al., 2021; Wolny et al., 2025). Therefore, there is an urgent need to monitor the dynamics, assess the degree of harm, prevent and mitigate HABs, especially in coastal areas closely related to human life and nature reserves.

Among over 2,800 dinoflagellate species (Guiry, 2024), Kareniaceae is one of the most important families responsible for HABs in the global coastal waters (Brand et al., 2012). The family Kareniaceae Bergholts, Daugbjerg, Moestrup and Fernández-Tejedor was established to accommodate *Karenia* G. Hansen and Moestrup, *Karlodinium* J. Larsen, and *Takayama* MF. Salas, Bolch, Botes and Hallegraeff, and three novel genera *Asterodinium* Sournia, *Gertia* K. Takahashi, G. Benico, Wai Mun Lum and M. Iwataki, and *Shimiella* Ok, H. J. Jeong, S. Y. Lee and Noh (Sournia, 1972; Takahashi et al., 2019; Ok et al., 2021) are also included in this family, which are characterized by unarmored, a straight or “s” shaped apical structure complex (ASC; = apical groove), and having fucoxanthin and/or fucoxanthin derivatives as their main accessory pigments (Bergholtz et al., 2006). *Karenia* is one of the most important genera, as most species in *Karenia* have been associated with fish kills and marine mammal mortality and can form toxic and harmful blooms (Brand et al., 2012). Among the eleven validly published *Karenia* species, seven of which have been shown to produce ichthyotoxins (Brand et al., 2012; Cen et al., 2024). *Karenia papilionacea* Haywood and Steidinger was first found in coastal waters of the east coast of the North Island and in the Foveaux Strait, New Zealand, and described as a novel species (Haywood et al., 2004). It was often present in the toxic blooms of *K. brevis*, and Haywood et al. communicated with Dr. Leanne Flewelling that *K. papilionacea* culture produced positive enzyme-linked immunosorbent assay (ELISA) results (50–110 pg.·mL^−1^) for brevetoxins. Three years later, Mooney et al. (2007) found that *K. papilionacea* can produce lipids, sterols, and PUFAs that are ichthyotoxic. However, the toxin identification was not confirmed by liquid chromatography-mass spectrometry (McNabb et al., 2012; Fowler et al., 2015). Fowler et al. (2015) isolated the toxic fraction of *K. papilionacea* culture and identified it as brevetoxin-2 (PbTx-2) using mass spectrometry and nuclear magnetic resonance, which confirmed that *K. papilionacea* is a toxic HAB species. In the recent 10 years, *K. papilionacea* has been reported in the coastal waters of Australia, New Zealand, Japan, Russia, France, China, South Korea, the USA, and Kuwait (Yamaguchi et al., 2016; Orlova et al., 2022; Kim et al., 2023; Cen et al., 2024; Wolny et al., 2024; Al-Kandari et al., 2025). Cen et al. (2024) found that exposure of *K. papilionacea* culture is toxic to marine medakas, *Oryzias melastigma*. In regard to the toxicity and wide distribution of *K. papilionacea*, it is crucial to further investigate the biology and ecology of this important HAB species.

In this study, we established a clonal culture of *K. papilionacea*, through single-cell isolation, from the coast of Qingdao (belonging to the Yellow Sea), China, and characterized its morphology using light microscopy (LM) and scanning electron microscopy (SEM), as well as its phylogeny based on large subunit (LSU) rDNA sequences. More importantly, we characterized the impact of *K. papilionacea* culture on brine shrimp egg hatching, as well as its toxicity to marine animals (rotifers, brine shrimp, and finfish) using laboratory bioassays. Our results provide a foundation for monitoring and risk assessment of this species in Chinese coastal waters and contribute essential knowledge to the fundamental ecology of *K. papilionacea*.

## 2 Materials and methods

### 2.1 Sample collection, culture establishment, and maintenance

A water sample (~1 m) was collected from the coast of Qingdao, Shandong province, China (36.00° N, 120.35° E) in September 2020 using a plankton net with a pore size of 20 μm. Single cells of dinoflagellate species were isolated using the micropipette technique and placed into individual wells of a 24 multi-well culture plate (Corning, Kennebunk, Maine, USA) containing 2.5 mL, 0.2 μm filter-sterilized and autoclaved natural seawater collected from the sampling station, and enriched with f/2-Si medium (Guillard, 1975) at 21 °C, and 12:12 h light: dark at ~100 μmol quanta m^−2^·s^−1^ supplied by white fluorescent lights. A strain of *K. papilionacea* (strain No. JZBD6-2020-3, referred to as Yellow Sea strain hereafter) was established and maintained in a 50 mL sterile tissue culture flask (Corning, Wujiang, Jiangsu, China), and was incubated under the same conditions.

### 2.2 Light microscopy and scanning electron microscopy

Live cells were visualized using a compound microscope (BX53, Olympus, Japan) equipped with a digital camera (DP80, Olympus, Japan) or Zeiss Imager A2 (Carl Zeiss, Gottingen, Germany) equipped with a camera (Axiocam 512 color, Carl Zeiss, Gottingen, Germany). Vegetative cells were observed under epifluorescence after staining live cultures with SYBR Green (Solarbio, Beijing, China), and photographed for chlorophyll-induced red autofluorescence and SYBR Green-induced green fluorescence of the nucleus. Cell sizes for 50 live cells at the mid-exponential growth phase were measured at × 200 magnification using a DP80 digital camera (Olympus, Tokyo, Japan).

Scanning electron microscopy (SEM) was completed according to our previous protocol (Hu et al., 2020a; Hu et al., 2020b). A 2 mL of aliquot of vegetative cells at mid-exponential growth stage was preserved with osmium tetroxide (OsO4, 2% final concentration) for 40–50 min. The cells were then gently filtered onto an 11-μm pore size Millipore nylon membrane and dehydrated in an acetone gradient (10, 30, 50, 70, 90%, and 3 × in 100%; for 15 min each). Filters were then critical point-dried with liquid CO_2_ (EM CPD300, Leica, Austria). Finally, the prepared filters were mounted on stubs, sputter-coated with gold–palladium (EM ACE200, Leica, Austria), and observed at 5 kV using a Scanning Electron Microscope (S-3400 N, Hitachi, Japan). Micrographs were processed with Adobe Photoshop 2021 (Adobe Inc., San Jose, California, USA) to overlay them uniformly on a black background.

### 2.3 DNA extraction, PCR amplification, and sequencing

Detailed methodological protocols for DNA extraction and sequencing of the *K. papilionacea* Yellow Sea strain were performed as previously described (Hu et al., 2021). Specifically, total genomic DNA was extracted from 10 mL of culture at the exponential growth stage using a plant DNA extraction kit (Tiangen, China) following the manufacturer’s protocol. Approximately 1,400 bp of large subunit (LSU) rDNA sequence was amplified using both primer set, D1R (F: 5’-ACCCGCTGAATTTAAGCATA-3′) (Scholin et al., 1994) and 28-1483R (R: 5′- GCTACTACCACCAAGATCTGC-3′) (Daugbjerg et al., 2000). The PCR reactions were conducted in a total volume of 20 μL, containing 7 μL of ddH_2_O, 10 μL of High Fidelity (HiFi) PCR SuperMix (TransGen, China), 1 μL of each PCR primer, and 1 μL of DNA template. The PCR protocol was as follows: initial denaturation at 94°C for 5 min, followed by 35 cycles at 94 °C for 30 s, 54 °C for 30 s, and 72 °C for 2 min, and extension for 10 min at 72 °C. PCR-amplified products were confirmed by 1.0% agarose gel electrophoresis. The amplicons were purified with the agarose gel DNA fragment recovery kit (GENEray, China) and ligated into the pMD-19 T vector (TaKaRa, Japan), and then transformed into *Escherichia coli* DH5α (Biomed, China). The positive clones were sequenced (Beijing Tsingke Biotech Co., Ltd., China). The new sequence was deposited in GenBank with accession number PV789634 (LSU rDNA).

### 2.4 Phylogenetic and genetic diversity analyses

Large subunit (LSU) rDNA gene sequences of *K. papilionacea* and other closely related Kareniaceae species were used for phylogenetic analysis. A sequence of *Gymnodinium catenatum* (accession No. AF200672) was used as the outgroup. Sequence alignments were performed using MAFFT v7.475 with the default settings^1^ (Katoh et al., 2019) and then modified with BioEdit v7.2.5 (Hall, 1999). The final alignment of LSU rDNA sequences included 750 aligned nucleotides, including gaps introduced by alignment. The TrN + I + G substitution model was selected as the best-fit model using jModelTest 2.1.4 (Darriba et al., 2012) based on the Akaike information criteria (Akaike, 1974). Phylogenetic trees were constructed using Bayesian inference (BI) and maximum likelihood (ML) analyses. The Bayesian inference (BI) analysis was performed with MrBayes 3.2.6 (Ronquist et al., 2012) using the best-fitting substitution model (TrN + I + G). Four independent Markov chain Monte Carlo simulations were run simultaneously for 10 million generations, and trees were sampled every 1,000 generations. The initial 25% of trees were discarded as burn-in, and convergence was judged based on the average standard deviation of split frequencies (all less than 0.01). The remaining trees were used to generate a consensus tree and calculate posterior probabilities for all branches using a majority-rule consensus approach. Maximum likelihood (ML) analysis was conducted with RaxML v7.2.6 (Stamatakis, 2006) using the model GTR + I + G (the model GTR + I + G ranked fourth, and the score of this model was close to model TrN + I + G), and node support was assessed with 1,000 bootstrap replicates. FigTree v1.4.4 was used to visualize the consensus tree.

The pairwise distances among the Kareniaceae species available in NCBI, our newly obtained sequence, and an entity annotated as *G. catenatum* (Accession No. AF200672) were computed. These sequences were aligned using MAFFT v7.475 with the default settings (see footnote 1) (Katoh et al., 2019) and modified manually using BioEdit v7.2.5 (Hall, 1999). The final alignment of LSU rDNA sequences included 750 aligned nucleotides, including gaps introduced by alignment. Pairwise evolutionary distances were then computed using the Jukes and Cantor algorithm implemented in MEGA 7.0 (Tamura et al., 2004; Kumar et al., 2016).

### 2.5 Influence of *Karenia papilionacea* on the hatching of brine shrimp (*Artemia salina*) eggs

In order to evaluate the effects of *K. papilionacea* on the hatching success of healthy eggs, exposure bioassays of brine shrimp (*Artemia salina*) eggs to whole-cell cultures at different cell densities were conducted. The brine shrimp eggs were purchased from Baofeng Biological Products Co., Ltd., China. A serial dilution (1.325 × 10^4^, 9.94 × 10^3^, 6.63 × 10^3^, 3.98 × 10^3^, and 1.33 × 10^3^ cells·mL^−1^) was performed on the culture of *K. papilionacea* at the exponential growth stage using f/2-Si medium (Guillard, 1975) and f/2-Si medium (0 cells·mL^−1^ of *K. papilionacea*) was used as a negative control. The hatching experiments were performed on 24 multi-well culture plates (Corning, Kennebunk, Maine, USA), with 2–3 resting eggs in each well and a total of 50 eggs in each plate. Each well contained 2.5 mL of test culture medium, which included a live cell culture of *K. papilionacea*. The egg hatching experiment was conducted at 21°C with an irradiance of 60 μmol photons m^−2^·s^−1^ and a 12:12 h light: dark photoperiod. The resting eggs were checked with an inverted microscope (AXIO Vert. A1, Zeiss, Oberkochen, Germany) every 24 h, and hatching rates were calculated at the incubation time points, e.g., 24 h, 48 h, 72 h, 96 h, and 120 h.

### 2.6 Exposure experiments of marine animals (rotifer, brine shrimp, and finfish)

In order to test the toxicity of *K. papilionacea* culture, exposure experiments of marine zooplankton (rotifer, *Brachionus plicatilis*; brine shrimp, *A. salina*) were conducted using the live cell culture of *K. papilionacea*. The resting eggs of rotifer *B. plicatilis* (L-type) and brine shrimp *A. salina* were bought from Ningbo Futian Biotechnology Co., Ltd. (China) and Baofeng Biological Products Co., Ltd. (China), respectively, and 2-day-old neonates were used in this study. Test animals were exposed to a serial dilution of live cell cultures (for *A. salina*: 1.325 × 10^4^, 9.940 × 10^3^, 6.630 × 10^3^, 3.980 × 10^3^, and 1.330 × 10^3^ cells·mL^−1^, respectively; for *B. plicatilis*, 1.0561 × 10^4^, 7.920 × 10^3^, 5.280 × 10^3^, and 2.640 × 10^3^ cells·mL^−1^, respectively), which were both obtained from diluting the initial culture (for *A. salina*: 1.325 × 10^4^ cells·mL^−1^, for *B. plicatilis*: 1.0561 × 10^4^ cells·mL^−1^, stationary growth stage) using f/2-Si medium. The f/2-Si medium and *Isochrysis galbana* (strain T-ISO, non-toxic prey) were used as negative controls. The bioassays were conducted in 24-well culture plates, with 2–3 test animals and 2.5 mL of pre-diluted culture added to each well (n = 50). Test animals were observed with an inverted microscope (AXIO Vert. A1, Zeiss, Oberkochen, Germany) every 12 h in the first 24 h and every 24 h after that within a period of 120 h, and animal death was recorded following complete cessation of locomotor activity. Dissolved oxygen (DO) levels of culture medium were measured at the beginning of the experiment and immediately after animal death, or at the end of the experiment.

For the toxicity of *K. papilionacea* on finfish, larval medaka *Oryzias melastigma* (3-day-old, ~3.5 mm in length) were exposed to a serial dilution of whole cell cultures, which were diluted from *K. papilionacea* culture at the stationary phase with a cell density of 1.093 × 10^4^ cells·mL^−1^ using f/2-Si medium. Briefly, ichthyotoxic experiments were conducted with 1–2 *O. melastigma* exposed to various concentrations of *K. papilionacea* (equivalent to 1.09 × 10^3^, 2.73 × 10^3^, 5.47 × 10^3^, 8.20 × 10^3^, and 1.093 × 10^4^ cells·mL^−1^) in 12-well culture plates (Corning, Kennebunk, Maine, USA), containing 5 mL of culture in each well (20 juveniles for each treatment). The f/2-Si medium and *I. galbana* (strain T-ISO, non-toxic prey) were used as negative controls. Finfishes were observed frequently in the first 24 h and every 24 h after that, within a period of 120 h to record immobilization and death time with an inverted microscope (AXIO Vert. A1, Zeiss, Oberkochen, Germany). Dissolved oxygen (DO) was measured before the addition of finfish, immediately after animal death, or at the termination of the experiment. All bioassay experiments were conducted under the same conditions as culture maintenance.

### 2.7 Statistics

In the toxicity experiments, differences among treatments were generally assessed using the t-test or one-way ANOVA followed by least significant difference (LSD) post-hoc test in the toxicity experiments. In all analyses, statistical significance was defined as a *p*-value of < 0.05 unless otherwise specified.

## 3 Results

### 3.1 Morphological and molecular confirmation of *Karenia papilionacea*

**Figure 1:**
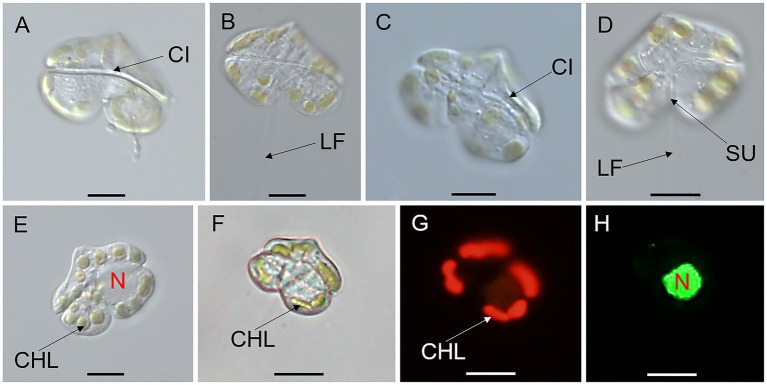
Micrographs (LM) of *Karenia papilionacea* Yellow Sea strain (strain No. JZBD6-2020-3). **(A,B)** The surface of the dorsal view shows the longitudinal flagellum (LF) and cingulum (CI). **(C,D)** The surface of the ventral view shows the cingulum (CI), sulcus (SU), and longitudinal and flagellum (LF). **(E)** Surface view shows 16 chloroplasts (CHL) and nucleus (N). **(F–H)** Bright and epifluorescence light microscopy observation of the same cell show chloroplasts (CHL) and a nucleus (N) located in the left lobe of the hypocone. Scale bars = 10 μm.

**Figure 2:**
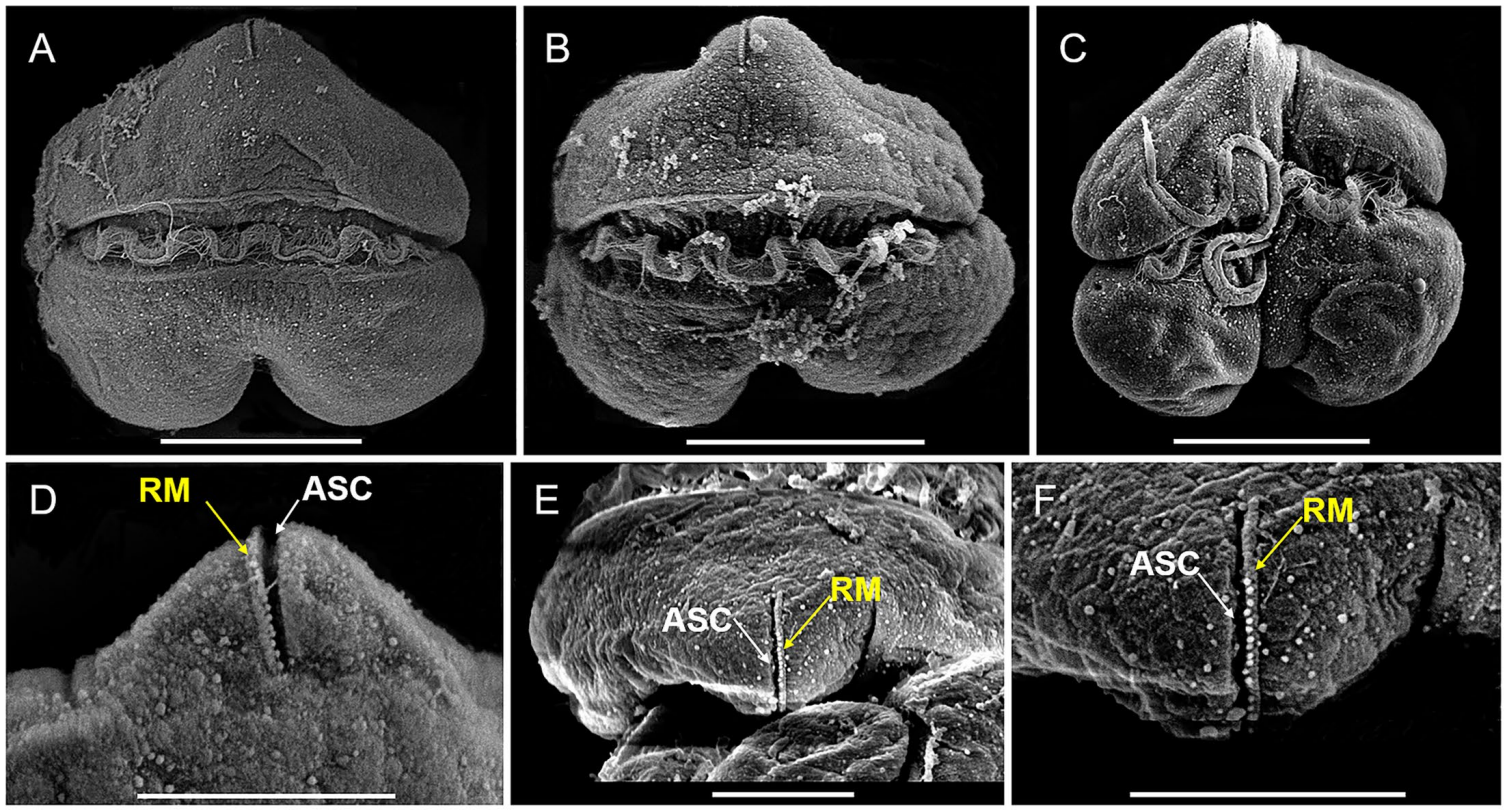
SEM micrographs of *Karenia papilionacea* Yellow Sea strain (strain No. JZBD6-2020-3). **(A,B)** Dorsal view shows the cingulum (CI) and transverse flagellum (TF). **(C)** Ventral view shows the cingulum (CI), sulcus (SU), and longitudinal and flagellum (LF). **(D)** Dorsal view shows the apical structure complex (ASC, white arrow) and rolled margin (RM, yellow arrow). **(E,F)** Different focus of the apical view shows the apical structure complex (ASC, white arrow) and rolled margin (RM, yellow arrow) of the same cell. Scale bars for **(A–C)** = 10 μm, **(D–F)** = 5 μm.

Vegetative cells of *K. papilionacea* Yellow Sea strain were 12.58–27.52 μm in length (20.71 ± 4.47 μm, *n* = 50) and 14.68–36.29 μm in width (26.73 ± 7.18 μm, *n* = 50), with a ratio of cell length to width of 0.63–0.96 (0.79 ± 0.09, *n* = 50; Figures 1, 2). The cells were solitary, transversely elongated, and dorsoventrally compressed (Figures 1, 2). The width was usually longer than the length, but under certain circumstances, the width was almost equal to its length (Figures 1, 2). The cells were divided into the epicone and hypocone by a deep cingulum (Figures 1, 2). The epicone possessed a pointed apical carina and a short linear apical structure complex (ASC) with rolled margins (Figures 1, 2). There was a rolled margin on the left side of the ASC (Figures 2D–F). The hypocone was bilobed and centrally excavated (Figures 1, 2D–F). The cingulum was deep and displaced by approximately one cingular width (Figures 1D, 2C). The sulcal intrusion extended onto the epicone (Figure 2C). The nucleus was spherical to slightly oval and located in the left lobe of the hypocone (Figures 1E,H). Two to 18 round or elongated chloroplasts of yellow-green color were located peripherally (Figure 1).

One partial LSU rDNA sequence of *K. papilionacea* was obtained from our clonal culture established on the coast of Qingdao, China. We compared the LSU rDNA sequence of our isolate (Yellow Sea strain; 1,539 bp, GenBank accession No. PV789634) with that of other strains of *K. papilionacea* in the NCBI database and found it was 97.77% (1,009 bp/1,032 bp) – 99.61% (1,533 bp/1,539 bp) identical to 60 entities annotated as *K. papilionacea*, with 97.77% to the South and East China Seas strains (strain Nos. HK-42 and PT-B1; GenBank accession Nos. PP801206, MT754557) and 99.61% to the French strain IFR562 (GenBank accession No. KJ508366), where there were 6–22 base differences (Supplementary Table S1). The LSU rDNA sequence (PV789634 (Yellow Sea strain)) was found to be 98.73% identical to an entity annotated as *Karenia* sp. (KJ508373), and 87.39–96.56% identical to other Kareniaceae species, indicating that *K. papilionacea* is conspecific with *Karenia* sp. (KJ508373) and distinct from other species (Table S1).

**Figure 3:**
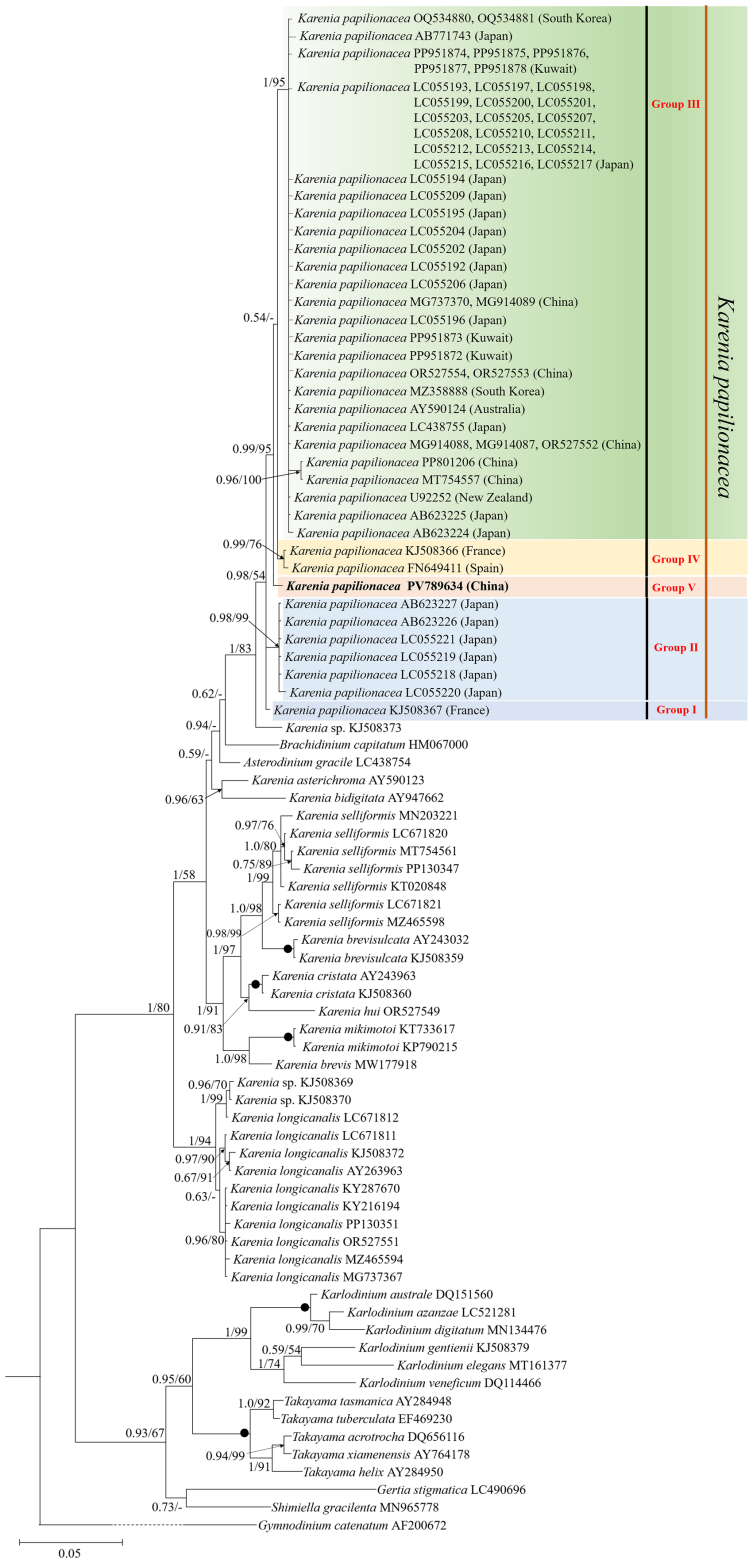
Molecular phylogeny of *Karenia papilionacea* and other Kareniaceae species inferred from partial large subunit rDNA (LSU rDNA) sequences using Bayesian inference (BI) with *Gymnodinium catenatum* (AF200672) as an outgroup. The new sequence of *K. papilionacea* (PV789634) is indicated in bold. Numbers on branches are statistical support values (left, Bayesian posterior probabilities; right, maximum likelihood (ML) bootstrap support values). Posterior probabilities (pp) above 0.5 and bootstrap values >50% are shown. Black circles (•) indicate maximal support (pp = 1.00 in BI and bootstrap support = 100% in ML, respectively). All branches are drawn to scale.

Phylogenetic analyses using Bayesian inference (BI) and maximum likelihood (ML) generated similar trees based on LSU rDNA sequences but differed on a few internal nodes (Figure 3). The newly sequenced *K. papilionacea* was placed in a well-supported clade of *K. papilionacea* and distinct from other described *Karenia* species (Figure 3). The clade of *K. papilionacea* was subdivided into five groups: group I consisted of an isolate from France, group II consisted only of six cultures from Japan, group III consisted of isolates from China, Japan, South Korea, Kuwait, Australia, and New Zealand, group IV consisted of isolates from France and Spain, and group V consisted of an isolate from China (Figure 3).

### 3.2 Genetic diversity

The Jukes–Cantor pairwise distance analysis revealed substantial genetic divergence among the studied taxa. Sequence divergence between Kareniaceae species and *G. catenatum* (AF200672) ranged from 0 to 2.853, while divergence within Kareniaceae species varied from 0 to 1.473. Notably, genetic distances among *K. papilionacea* isolates ranged from 0.000 to 0.011 (Supplementary Table S2), yet these findings collectively underscored the high genetic diversity within this species.

### 3.3 Effect of *Karenia papilionacea* on hatching of brine shrimp eggs

**Figure 4:**
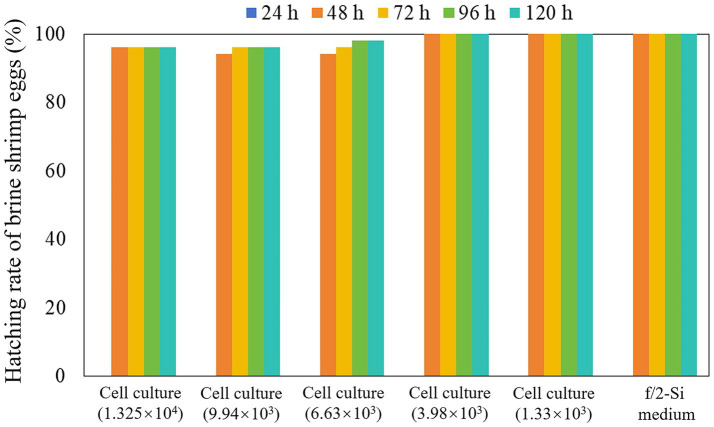
Hatching rate of brine shrimp eggs in the live cell culture of *Karenia papilionacea* Yellow Sea strain (Strain No. JZBD6-2020-3) with a series of cell densities within 120 h. F/2-Si medium was used as a negative control.

Across all experimental groups and the control group, no brine shrimp egg hatching (0% hatching rate) was observed within the 24-h incubation period (Figure 4). After 48 h, the hatching rate increased significantly, exceeding 90% in both experimental and control groups, and a 100% hatching rate in the control group as well as in treatment groups exposed to *K. papilionacea* at concentrations of 1.33 × 10^3^ and 3.98 × 10^3^ cells·mL^−1^ (Figure 4). After 72 h of incubation, the cumulative hatching rate surpassed 96% across all experimental groups (Figure 4). From 96 to 120 h, hatching rates remained stable with no significant change compared to the 72-h time point, consistently exceeding 96% across all experimental groups; only 1–2 brine shrimp eggs were not hatched in treatment groups exposed to *K. papilionacea* at concentrations of 6.63 × 10^3^, 9.94 × 10^3^, and 1.325 × 10^4^ cells·mL^−1^ (Figure 4).

### 3.4 Toxicity of *Karenia papilionacea* to rotifer, brine shrimp, and larval finfish

**Figure 5:**
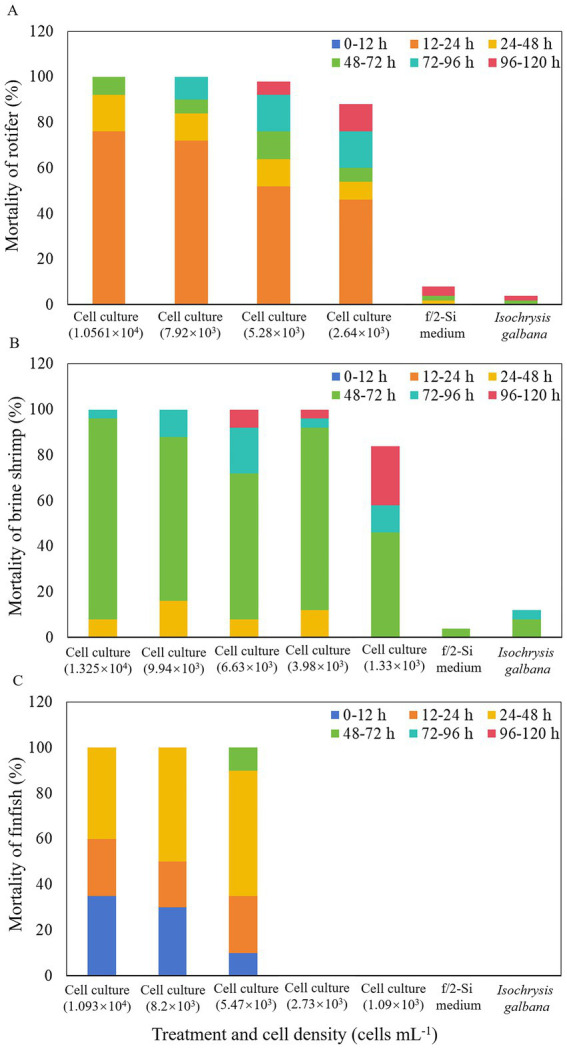
Mortality of rotifer *Brachionus plicatilis* **(A)**, brine shrimp *Artemia salina* **(B)**, and finfish marine medaka *Oryzias melastigma* **(C)** treated with a density range of live cells of *Karenia papilionacea* Yellow Sea strain (strain No. JZBD6-2020-3) within 120 h. F/2-Si medium and *Isochrysis galbana* (T-ISO, non-toxic) were used as negative controls.

The whole cell culture of *K. papilionacea* demonstrated potent toxic effects against two zooplankton species, rotifer *B. plicatilis*, and brine shrimp *A. salina*, and larval finfish *O. melastigma* (Figure 5). In all experiments, dissolved oxygen (DO) levels were maintained above 70% of saturation at 21 ± 1 °C, and animal mortalities did not exceed 12% in the controls. During the first 12 h of exposure, no rotifer mortality was observed. However, within 24 h, *B. plicatilis* exhibited significantly higher mortality (46–76%) in live cell cultures at densities of 2.64 × 10^3^–1.0561 × 10^4^ cells·mL^−1^ (*p* < 0.05; Figure 5A). After 48-h exposure, the mortality rates of rotifers increased to 54–64% at the lower algal cell densities (2.64 × 10^3^–5.28 × 10^3^ cells·mL^−1^) and 84–92% at the higher algal cell densities (7.92 × 10^3^–1.0561 × 10^4^ cells·mL^−1^). At 120-h exposure, 100% mortality rates of rotifers were observed at the two highest cell densities of 7.92 × 10^3^ and 1.0561 × 10^4^ cells·mL^−1^, and 88–98% at the two lower cell densities of 2.64 × 10^3^ and 5.28 × 10^3^ cells·mL^−1^ (Figure 5A).

For brine shrimp bioassays, no mortality of *A. salina* was observed after 24 h in all experiments. However, after 48 h of exposure to live *K. papilionacea* cell cultures (1.33 × 10^3^–1.325 × 10^4^ cells·mL^−1^), mortality rates increased to 8–16% (Figure 5B). At the time point of 72 h, the immobilization rates increased significantly to 46–96% in live cell cultures (*p* < 0.05; Figure 5B). After 96-h exposure, 100% mortality rates of brine shrimps were observed at the two highest cell densities of 9.94 × 10^3^ and 1.325 × 10^4^ cells·mL^−1^, and 58–96% at the three lower cell densities (1.33 × 10^3^ and 6.63 × 10^3^ cells·mL^−1^; Figure 5B). Mortalities of brine shrimps on 120 h exposure were 100% at cell densities ranging from 3.98 × 10^3^ to 1.325 × 10^4^ cells·mL^−1^, and 84% at the lowest cell density (1.33 × 10^3^ cells·mL^−1^; Figure 5B).

In the finfish exposure experiment, no larval *O. melastigma* died when exposed to live *K. papilionacea* cell cultures with cell densities less than 2.73 × 10^3^ cells·mL^−1^ during the 120-h exposure (Figure 5C). During the first 12 h of exposure, 10–35% fish mortality rates were observed in live cell cultures (5.47 × 10^3^–1.093 × 10^4^ cells·mL^−1^). After 48-h exposure, 100% mortality rates of larval finfish were observed at the two highest cell densities of 8.20 × 10^3^ and 1.093 × 10^4^ cells·mL^−1^ and 90% at 5.47 × 10^3^ cells·mL^−1^ (Figure 5C). At the time point of 72 h, the immobilization rates increased to 100% in live cell cultures with a cell density of 5.47 × 10^3^ cells·mL^−1^ (Figure 5C).

## 4 Discussion

### 4.1 Morphological and molecular characterization

The adverse effects of *Karenia* species on aquatic animals have been widely documented in various regions (Brand et al., 2012; Baldrich et al., 2024; Cen et al., 2024; Zhang et al., 2025). Given their ecological and economic impacts, accurate species identification is essential for effectively monitoring the dynamics of *Karenia* blooms and tracking the spread of toxic populations (Kim et al., 2023; Al-Kandari et al., 2025). Due to the high morphological similarity among morphotypes and their inherent plasticity, detailed morphological characterization is essential for distinguishing *Karenia* species. *Karenia* species are characterized as unarmored (or naked), having a linear apical structure complex (or apical groove), and fucoxanthin, or butanoyl-oxyfucoxanthin as major accessory pigments (Daugbjerg et al., 2000; Brand et al., 2012). *Karenia* blooms generally contain one *Karenia* species as the dominant species, but other *Karenia* species are also abundantly present (Heil and Steidinger, 2009; Kwok et al., 2016; Wang et al., 2023; Merino-Virgilio et al., 2012). Notably, *K. papilionacea* frequently co-occurs with *K. brevis* during HAB events (Merino-Virgilio et al., 2012). *Karenia papilionacea* exhibits morphological similarities to *K. brevis* but can be distinguished based on characteristics such as cell morphology, size (length and width), and the length of the apical structure complex (ASC) (Haywood et al., 2004). Furthermore, the occurrence of two distinct cell forms (large and small) has been documented in *K. papilionacea* (Fowler et al., 2015; Wolny et al., 2024). Due to these morphological overlaps and complexities, *K. papilionacea* has been frequently misidentified as *K. brevis* in previous studies (Al-Kandari et al., 2025). We reported *K. papilionacea* in the northern part of China (Yellow Sea) for the first time; however, it has been reported in the East and South China Seas (Cen et al., 2024). The diagnostic characters of our strain were in accordance with other strains (Haywood et al., 2004; Cen et al., 2024). Although the diagnostic characters of *K. papilionacea* are discernible through LM and SEM of well-preserved samples, accurate identification based on morphology demands an experienced researcher. This dependency on expert judgment limits practicality. Molecular-based methods (e.g., real-time PCR, amplicon sequencing, and fluorescence *in situ* hybridization) thus offer a more robust solution, enabling rapid, sensitive, and accurate detection of *Karenia* species directly in the field (Yamaguchi et al., 2016; Kim et al., 2023).

Based on the morphological and molecular phylogenetic (LSU rDNA and ITS regions) examinations, *K. papilionacea* has been divided into two phylotypes (original phylotype and phylotype-I), which displayed the same morphological characteristics (Yamaguchi et al., 2016). Wang et al. (2023) divided *K. papilionacea* into four clades based on the ITS region, with clades I and II belonging to the original phylotype and clades III and IV to phylotype-I. In this study, we found that *K. papilionacea* included five groups. Groups III, IV, and V (obtained in this study) belonged to the original phylotype, while phylotype-I included in groups I and II. The strains in groups I and IV were isolated from the coastal waters of Europe, six strains within group II from Asia (Japan), one strain in group V from Asia (China), and the remaining strains in group III from Asia (China, Japan, South Korea, and Kuwait) and Oceania (Australia and New Zealand). We found that the Yellow Sea strain belongs to a new group, and the genetic distance between the Yellow Sea strain and other *K. papilionacea* isolates ranged from 0.002 to 0.011, corresponding to 6–23 base differences. It is noteworthy that the Chinese strains belonged to groups III and V, while the Japanese strains belonged to groups II and III. There are only 1–2 strains in groups I, IV, and V. More information (e.g., rDNA sequences of more isolates in different groups) is needed to assess the relationship between biogeography and groups.

### 4.2 Toxicity of *Karenia papilionacea* and its ecological implications

Many *Karenia* species, such as *K. brevis*, *K. mikimotoi*, *K. bicuneiformis*, *K. brevisulcata*, *K. concordia*, *K. cristata*, and *K. selliformis,* have been reported to produce ichthyotoxins, e.g., brevetoxins (BTXs), gymnocins, gymnodimines (GYMs), and brevisulcatic acids (Brand et al., 2012; Cen et al., 2024). Fowler et al. (2015) detected brevetoxin-2 in *K. papilionacea* cultures isolated from coastal Delaware, USA. Subsequently, Basti et al. (2015) have reported that a strain of *K. papilionacea* exhibits negative effects on Japanese pearl oysters *Pinctada fucata martensii*. Recently, Wang et al. (2023) reported a fish-killing bloom dominated by four Kareniaceae species (*K. longicanalis*, *K. papilionacea*, *Karlodinium veneficum*, and *K. digitatum*) that strongly inhibited the swimming capacities and survival of two zooplankton species (*B. plicatilis* and *A. salina*). Cen et al. (2024) found that three strains of *K. papilionacea* cultures from the East and South China Seas were capable of producing gymnodimine-A (GYM-A) and were lethal to 60-day-old marine medakas. Therefore, *K. papilionacea* should be characterized as a toxic HAB-forming species.

In the past, the impacts of toxic algae on the hatching of zooplankton eggs were poorly understood. Song et al. (2021) found that hatching success of healthy rotifer eggs was observed to be significantly influenced by the live cell cultures and cell-free culture media of *Alexandrium insuetum*, which produces toxins that are neither PSTs nor spiroimines (13-desmethyl spirolide C and gymnodimine). Here, we found that the Yellow Sea strain of *K. papilionacea* from China, had minor impacts on the hatching of brine shrimp eggs, only 2–4% (1–2 eggs) not hatching in the treatments with higher cell densities (6.63 × 10^3^ cells·mL^−1^–1.325 × 10^4^ cells·mL^−1^), and 100% hatching in the treatments with lower cell densities (1.33 × 10^3^ cells·mL^−1^–3.98 × 10^3^ cells·mL^−1^) and the control. The cyst shell of the brine shrimp is composed of a triple-layered structure (Sugumar and Munuswamy, 2006; Li et al., 2024), while the rotifer resting egg is protected by a relatively simple shell primarily made of a chitin-protein composite (Munuswamy et al., 1996). We propose that the robust, multilayered cyst shell of the brine shrimp provides an effective barrier against external toxins, whereas the simple structure of the rotifer resting egg offers comparatively less protection. Brine shrimp eggs (dormant cysts) are indeed fundamental to the persistence, dispersal, and ecological function of brine shrimp (*Artemia*) populations. From our data, it is suggested that the *K. papilionacea* bloom has a minor impact on the hatching of brine shrimp eggs.

Our results clearly showed that *K. papilionacea* culture exhibited significant toxicity to rotifer, brine shrimp, and larval marine medakas. More than 50% of mortality was observed within the first 48 h for rotifers, 72 h for brine shrimp, and 48 h for larval marine medakas. For the marine medakas, all died in the first 48 h for the two treatments with higher cell densities and 72 h for the treatment with 5.47 × 10^3^ cells·mL^−1^, but for the two treatments with lower cell densities (1.09 × 10^3^ cells·mL^−1^–2.73 × 10^3^ cells·mL^−1^), all finfish were still alive in the 120-h incubation. Cen et al. (2024) found that 86.67% of marine medakas (*O. melastigma*, 60-day-old) died when exposed to a strain of *K. papilionacea* culture (at a density of 3.00 × 10^4^ cells·mL^−1^) from Tolo Harbour, South China Sea. It seems that larval *O. melastigma* is more sensitive to *K. papilionacea*. Besides, our strain belongs to group V, the strain from Tolo Harbour, South China Sea group III, maybe they are different ecotypes. *Karenia papilionacea* blooms have been reported in the field (Wang et al., 2023; Wolny et al., 2025). Wang et al. (2023) found that bloom water (dominant species *K. papilionacea*, *K. longicanalis*, *K. veneficum*, and *K. digitatum*) strongly inhibited the swimming capacities and survival of *B. plicatilis* and *A. salina*; however, the toxins (e.g., brevetoxin or other ichthyotoxic compounds) have not been determined. Furthermore, the toxicity difference between the strains from the Yellow Sea and South China Sea will be resolved by the quality of the lipophilic toxins. Our results revealed that *K. papilionacea* exerted lethal effects on aquatic organisms at various trophic levels, providing scientific evidence for evaluating the negative impacts of algal blooms on zooplankton communities and the marine ecosystem.

## 5 Conclusion

This study presents the first report of *K. papilionacea* from the northern part of China, which broadens the geographical distribution of this toxic HAB-forming species. We also characterized the morphology, molecular phylogeny, and toxicology of *K. papilionacea*. This strain belongs to a new group, which is different from other strains reported in the East and South China Seas. The Yellow Sea strain exhibited significant lethal effects on rotifer, brine shrimp, and finfish, but had a minor impact on the hatching success of brine shrimp eggs. Our observations and investigations provide a foundation for monitoring and risk assessment of *K. papilionacea* in Chinese coastal waters and contribute essential knowledge to the fundamental ecology of this toxic species. Further studies will be required to identify and qualify the toxin(s) of *K. papilionacea*.

## Data Availability

The datasets presented in this study can be found in online repositories. The names of the repository/repositories and accession number(s) can be found in the article/Supplementary material.

## Footnotes

^1^ http://mafft.cbrc.jp/alignment/server/
