# Supplementary material for: Examination of the toxicity of a new group of Karenia papilionacea isolated from the Yellow Sea, China, to multiple species of marine aquatic animals
Source: Front Microbiol. 2025 Nov 6;16:1687096. doi: 10.3389/fmicb.2025.1687096 (PMC12631269; doi:10.3389/fmicb.2025.1687096)
Supplement: Supplementary file 1 [file Table_1.docx]

Table S1 Comparisons of the rDNA sequence of *Karenia papilionacea* (1,539 bp, GenBank accession No. PV789634) with that of other species in Kareniaceae in NCBI database

| Species and GenBank accession number | Identity |
| --- | --- |
| *Karenia papilionacea* (OQ534881) | 99.02% (708 bp/715 bp) |
| *Kare. papilionacea* (OQ534880) | 99.03% (711 bp/718 bp) |
| *Kare. papilionacea* (AB771743) | 98.61% (638 bp/647 bp) |
| *Kare. papilionacea* (PP951878) | 99.01% (700 bp/707 bp) |
| *Kare. papilionacea* (PP951877) | 99.01% (703 bp/710 bp) |
| *Kare. papilionacea* (PP951876) | 99.01% (701 bp/708 bp) |
| *Kare. papilionacea* (PP951875) | 99.01% (701 bp/708 bp) |
| *Kare. papilionacea* (PP951874) | 99.01% (701 bp/708 bp) |
| *Kare. papilionacea* (LC055217) | 99.08% (756 bp/763 bp) |
| *Kare. papilionacea* (LC055216) | 99.08% (756 bp/763 bp) |
| *Kare. papilionacea* (LC055215) | 99.08% (756 bp/763 bp) |
| *Kare. papilionacea* (LC055214) | 99.08% (756 bp/763 bp) |
| *Kare. papilionacea* (LC055213) | 99.08% (756 bp/763 bp) |
| *Kare. papilionacea* (LC055212) | 99.08% (756 bp/763 bp) |
| *Kare. papilionacea* (LC055211) | 99.08% (756 bp/763 bp) |
| *Kare. papilionacea* (LC055210) | 99.08% (756 bp/763 bp) |
| *Kare. papilionacea* (LC055208) | 99.08% (756 bp/763 bp) |
| *Kare. papilionacea* (LC055207) | 99.08% (756 bp/763 bp) |
| *Kare. papilionacea* (LC055205) | 99.08% (756 bp/763 bp) |
| *Kare. papilionacea* (LC055203) | 99.08% (756 bp/763 bp) |
| *Kare. papilionacea* (LC055201) | 99.08% (756 bp/763 bp) |
| *Kare. papilionacea* (LC055200) | 99.08% (756 bp/763 bp) |
| *Kare. papilionacea* (LC055199) | 99.08% (756 bp/763 bp) |
| *Kare. papilionacea* (LC055198) | 99.08% (756 bp/763 bp) |
| *Kare. papilionacea* (LC055197) | 99.08% (756 bp/763 bp) |
| *Kare. papilionacea* (LC055195) | 99.08% (756 bp/763 bp) |
| *Kare. papilionacea* (LC055194) | 99.08% (756 bp/763 bp) |
| *Kare. papilionacea* (LC055193) | 99.08% (756 bp/763 bp) |
| *Kare. papilionacea* (LC055209) | 99.08% (756 bp/763 bp) |
| *Kare. papilionacea* (LC055204) | 98.95% (756 bp/764 bp) |
| *Kare. papilionacea* (LC055202) | 98.95% (756 bp/764 bp) |
| *Kare. papilionacea* (LC055192) | 98.82% (754 bp/763 bp) |
| *Kare. papilionacea* (LC055206) | 98.82% (755 bp/764 bp) |
| *Kare. papilionacea* (MG737370) | 99.08% (732 bp/739 bp) |
| *Kare. papilionacea* (MG914089) | 99.00% (691 bp/698 bp) |
| *Kare. papilionacea* (LC055196) | 98.56% (753 bp/764 bp) |
| *Kare. papilionacea* (PP951873) | 98.92% (828 bp/837 bp) |
| *Kare. papilionacea* (PP951872) | 98.94% (932 bp/942 bp) |
| *Kare. papilionacea* (OR527554) | 99.05% (835 bp/843 bp) |
| *Kare. papilionacea* (OR527553) | 99.08% (886 bp/874 bp) |
| *Kare. papilionacea* (MZ358888) | 99.02% (911 bp/920 bp) |
| *Kare. papilionacea* (AY590124) | 98.99% (882 bp/891 bp) |
| *Kare. papilionacea* (LC438755) | 99.09% (882 bp/891 bp) |
| *Kare. papilionacea* (MG914088) | 99.07% (960 bp/969 bp) |
| *Kare. papilionacea* (MG914087) | 99.03% (922 bp/931 bp) |
| *Kare. papilionacea* (OR527552) | 99.04% (828 bp/838 bp) |
| *Kare. papilionacea* (PP801206) | 97.77% (1009 bp/1032 bp) |
| *Kare. papilionacea* (MT754557) | 97.77% (1009 bp/1032 bp) |
| *Kare. papilionacea* (U92252) | 98.76% (715 bp/724 bp) |
| *Kare. papilionacea* (AB623225) | 98.69% (756 bp/766 bp) |
| *Kare. papilionacea* (AB623224) | 98.57% (756 bp/767 bp) |
| *Kare. papilionacea* (KJ508366) | 99.61% (1533 bp/1539 bp) |
| *Kare. papilionacea* (FN649411) | 99.31% (718 bp/723 bp) |
| *Kare. papilionacea* (AB623227) | 98.56% (753 bp/764 bp) |
| *Kare. papilionacea* (AB623226) | 98.82% (754 bp/763 bp) |
| *Kare. papilionacea* (LC055221) | 98.82% (754 bp/763 bp) |
| *Kare. papilionacea* (LC055219) | 98.82% (754 bp/763 bp) |
| *Kare. papilionacea* (LC055218) | 98.82% (754 bp/763 bp) |
| *Kare. papilionacea* (LC055220) | 98.43% (752 bp/764 bp) |
| *Kare. papilionacea* (KJ508367) | 99.30% (990 bp/997 bp) |
| *Karenia* sp. (KJ508373) | 98.73% (1475 bp/1494 bp) |
| *Kare. mikimotoi* (KT733617) | 92.77% (680 bp/733 bp) |
| *Karl. veneficum* (DQ114466) | 90.95% (1307 bp/1437 bp) |
| *Takayama tasmanica* (AY284948) | 87.39% (790 bp/904 bp) |
| *Brachidinium capitatum* (HM067000) | 94.68% (658 bp/695 bp) |
| *Asterodinium gracile* (LC438754) | 96.56% (954 bp/988 bp) |
| *Gertia stigmatica* (LC490696) | 90.91% (1400 bp/1540 bp) |
| *Shimiella gracilenta* (MN965778) | 88.18% (828 bp/939 bp) |
| *Gymnodinium catenatum* (AF200672) | 80.34% (809 bp/1007 bp) |
